# Supplementary material for: Brucella abortus histidine auxotrophs are copper sensitive
Source: J Bacteriol. 2026 Feb 4;208(3):e00492-25. doi: 10.1128/jb.00492-25 (PMC13001215; doi:10.1128/jb.00492-25)
Supplement: Supplemental tables and figures — Tables S1 to S5 and Figures S1 to S4. [file jb.00492-25-s0001.pdf]

Supplementary information

For Focant *et al.* "*Brucella abortus* histidine auxotrophs are copper sensitive"

Content :

Table S1 | Histidine enrichment in *Brucella abortus* strain 2308 proteome

Table S2 | Suppressor mutations

Table S3 | List of primers used in this study

Table S4| List of plasmids used in this study.

Table S5| List of strains used in this study.

Figure S1 | Schematic representation of the histidine biosynthesis pathway

Figure S2 | Histidine auxotroph mutants are able to grow in a rich medium

Figure S3 | Histidine auxotroph mutants are copper sensitive

Figure S4 | Suppressive mutation does not save histidine auxotrophy.

Table S1. Histidine enrichment in *Brucella abortus* strain 2308 proteome

Locus_tag	Histidine percentage (%)	Name	Function
BAB1_0301	14.0	UreE	Nickel donor during urease metallocentre assembly
BAB1_1974	10.7	QueD	7-cyano-7-deazaguanine biosynthesis and purine metabolism
BAB_RS33715	10.5	Hypothetical protein	Unknown
BAB_RS22295	9.7	CbtB	Cobalt transporter subunit
BAB2_0432	9.1	NikR	Transcriptional repressor of the *nikABCDE* operon
BAB_RS32835	9.1	Putative CTP synthase	Unknow
BAB2_0246	8.4	GTP-binding protein	Cobalamin synthesis
BAB2_1080	7.7	ZnuC	Energy coupling in ZnuABC zinc uptake transport system
BAB1_2027	7.7	BolA-like protein	DNA-binding regulator stress-induced morphogen
BAB1_1818	7.4	Usg protein	Unknown
BAB2_0964	7.3	GAF/GGDEF domain protein	Diguanylate cyclase
BAB_RS33725	7.3	Hypothetical protein	Unknown
BAB1_1173	7.2	LpxA	Lipid A biosynthesis
BAB1_0771	7.1	Zinc finger domain	Unknown
BAB1_0174	7.1	Lactoylglutathione lyase	Methylglyoxal detoxification
BAB2_0545	7.0	RibH2	Riboflavin biosynthesis
BAB1_1743	6.9	Hypothetical protein	Unknown
BAB1_2036	6.9	GTP-binding protein	Cobalamin synthesis protein
BAB1_2175	6.9	Irr	Ferric uptake regulation
BAB_RS25890	6.9	Hypothetical protein	Unknown
BAB_RS21185	6.8	Hypothetical protein	Unknown
BAB1_1754	6.8	YdcH family protein	Unknown
BAB1_1347	6.7	Serine hydrolase (ydeN)	Esterase activity
BAB_RS17660	6.6	Superoxyde dismutase	Superoxyde dismutase activity
BAB_RS22375	6.5	Serine hydrolase family protein	Esterase activity
BAB1_1991	6.5	Gamma-glutamylcyclotransferase	Glutathione catabolic process
BAB_RS17240	6.4	Uncharacterized protein	Unknown
BAB1_1668	6.4	Fur	Ferric uptake negative regulation
BAB1_1476	6.4	Alpha-beta hydrolase	Lipid transport and metabolism
BAB2_0535	6.4	SodC	Superoxide dismutase activity; radicals elimination
BAB2_1079	6.3	ZnuA	ZnuABC zinc uptake transport system
BAB_RS32250	6.25	Uncharacterized protein	Unknown
BAB2_1082	6.2	Zur	Fe^{2+} or Zn^{2+} uptake regulation
BAB1_0161	6.2	DUF1150 family protein	Unknown
BAB1_0497	6.2	Cytochrome c oxydase III	Aerobic electron transport chain
BAB1_0751	6.2	Uncharacterized protein	Unknown
BAB2_0644	5.9	Metal-dependent hydrolase	Hydrolase activity
BAB1_0714	5.9	Dommage-inducible protein DinB	Unknown

BAB1_0518	5.9	Adenine DNA glycosylase	Adenine glycosylase active on G_A mispairs
BAB1_0556	5.8	Uncharacterized protein	Unknown
BAB1_0042	5.8	cytochrome o ubiquinol oxidase subunit IV	Electron transport
BAB1_1716	5.8	RuvC	Nuclease activity; Holliday junction intermediates resolution
BAB1_0456	5.7	Histone deacetylase family	Deacetylase activity
BAB1_1899	5.7	VOC family protein	Glyoxalase/Bleomycin resistance protein/dioxygenase
BAB1_1689	5.6	Uncharacterized protein	Unknown
BAB1_1732	5.6	2Fe-2S ferredoxins, iron-sulfur binding protein	Unknown
BAB1_1828	5.6	Uncharacterized protein	Unknown
BAB1_1189	5.6	Uncharacterized protein	Unknown
BAB2_0495	5.6	Uncharacterized protein	Unknown
BAB_RS22085	5.5	Uncharacterized protein	Unknown
BAB1_1001	5.5	Uncharacterized protein	Unknown
BAB1_1755	5.5	Uncharacterized protein	Unknown
BAB2_1068	5.5	Class II aldolase/adducin, N-terminal:ATP/GTP-binding site	Carbohydrate transport and metabolism
BAB2_1145	5.5	Sulfatase	Sulfuric ester hydrolase activity
BAB2_0505	5.4	Lectin-like protein BA14k	Immunoglobulin-binding and hemagglutination properties virulence (LPS biosynthesis)
BAB2_0534	5.4	CueO	Copper oxidation
BAB2_1016	5.4	Universal stress protein (Usp):Usp domain	Stress survival
BAB1_0814	5.4	Uncharacterized protein	Unknown
BAB1_0135	5.4	Uncharacterized protein	Unknown
BAB1_1617	5.3	Gfo/Idh/MocA family oxidoreductase	Dehydrogenase activity
BAB1_1581	5.3	Metallophosphoesterase	Hydrolase activity
BAB1_1297	5.3	DUF2218 domain-containing protein	Unknown
BAB1_1495	5.3	Antifreeze protein, type I	Subzero environments survival
BAB1_1904	5.3	GCN5-related N-acetyltransferase	Acetyltransferase activity
BAB1_0041	5.3	Cytochrome o ubiquinol oxidase subunit III	Aerobic electron transport chain
BAB1_1568	5.3	Aspartyl/asparaginyl beta-hydroxylase domain-containing protein	Unknown
BAB1_0573	5.2	Helix-turn-helix transcriptional regulator	Transcriptional regulator
BAB1_0856	5.2	BolA-like protein	DNA-binding regulator stress-induced morphogen
BAB1_1666	5.2	Pseudouridine synthase	Pseudourine synthesis from uracil
BAB1_1284	5.2	Cold-shock DNA-binding domain	DNA-binding in low temperature
BAB1_1277	5.2	PAS domain-containing protein	Unknown
BAB1_1219	5.2	DJ-1/PfpI family protein	Peptidase activity
BAB1_2161	5.2	TrmB	N(7)-methylguanine tRNA

			biosynthesis
BAB1_1632	5.1	Uncharacterized protein	Unknown
BAB1_0085	5.1	YncA	Amino acid transport and metabolism
BAB2_0777	5.1	GntR family	DNA-binding transcriptional regulator
BAB2_0326	5.1	PurU	IMP biosynthesis via de novo pathway
BAB1_0926	5.1	AroQ	Chorismate biosynthesis
BAB1_0051	5.1	DUF1775 domain-containing protein	Unknown
BAB_RS33345	5.1	Uncharacterized protein	Unknown
BAB2_0897	5.1	Oxidoreductase domain	Dehydrogenase activity
BAB2_0170	5.1	YdcH family protein	Unknown
BAB1_1355	5.0	Calcium-binding EF-hand	Calcium binding
BAB1_0961	5.0	Uncharacterized protein	Unknown
BAB2_0802	5.0	PIG-L family deacetylase	Deacetylase activity

Proteome fasta file was downloaded from NCBI and submitted by Oak Ridge National Laboratory. The bioinformatic programme used was made by Damien Devos (CABD, Sevilla, Spain). It reads the fasta file and, for each protein sequence, counts the number of the amino acid histidine (represented by an H in the sequence). It also calculates the percentage of histidine in the sequence in order to illustrate the enrichment of proteins in histidine.

[a] % indicates the percentage of histidine in the amino acid sequence of the protein.

[b] We have established an arbitrary threshold value of 5 %, proteins with a percentage equal to or greater than 5 are found in the table above. A minority of the protein has enrichment above 5 % and these proteins could be more strongly impacted by histidine starvation caused by mutations in the biosynthetic pathway. Only 85 out of 2941 predicted proteins (thus 2.9%) have a proportion of His residues >5%.

Table S2. Suppressor mutations

Mutant[a]	Suppressor[b]	Position[c]	Type[d]	Ref.[e]	Alt.[f]	Locus_tag[g]		Name[h]
Δ*hisA*	A1	chr1. 1472668	SNP	G	T	BAB_v1_a1531	BAB1_1517	LysR21
	A2	chr1. 936216	Del.	GT	G	BAB_v1_a0971 & BAB_v1_a0972	BAB1_0955 & 0956	Intergenic region
Δ*hisD*	D4	chr1. 1124993	Del.	AG	A	BAB_v1_a1169	BAB1_1152	PdhA
	D6	chr1. 1124208	SNP	G	T	BAB_v1_a1168	BAB1_1151	PdhB

[a] Parental mutant strain
[b] Suppressor number
[c] Position of mutation on chromosome (chr) 1 or 2
[d] Type of mutation: Del=deletion, SNP= single nucleotide polymorphism
[e] Reference genome nucleotide
[f] Suppressor alternative nucleotide
[g] The coding sequences (ORFs) in *B. abortus* 544 (left) and their correspondence in *B. abortus* 2308 (right)
[h] Gene name

Table S3. List of primers used in this study. Nucleotides not annealing on DNA template are shown in lowercase, red characters represent point mutations and restriction sites are underlined.

Primer name	5'-3' sequence
**F1-copA-bis**	CCCTATCGGTGATTGGGTC
**R1-copA-bis**	gctcagaacgCATGGCAATCCCTTTCGTG
**F2-copA-bis**	gattgccatgCGTTCTGAGCAATGCCTTG
**R2-copA**	GAAGCGAACAGGTCGAAC
**F1-cueO**	GTGAAAGGGGGCTTTCTC
**R1-cueO**	aaacggtcacGGCGGGTAATTCCAGTCATG
**F2-cueO**	attacccgccGTGACCGTTTGAGAGCAAGG
**R2-cueO**	AAGAGCCGGTACGATCTTGC
**F1-hisA**	CCCATGAAAGCGGCATATCG
**R1-hisA-bis**	catcgtaaagTTGAGGCGCACGCATTGAC
**F2-hisA-bis**	tgcgcctcaaCTTTACGATGGCCGCATCG
**R2-hisA**	TGCTATCAACGGACCGGATCG
**F-compl-hisA-Xba1**	ctagtctagaTCGGCCTTTCGCTCATTGC
**R-compl-hisA-BamH1**	acgcggatccGCGGCCATCTTTCACATCGAG
**F1-hisB**	CGTAATTGCCGCCGGAAC
**R1-hisB**	cttgagcgacCAGCGGTCATAGGCTTTCC
**F2-hisB**	atgaccgctgGTCGCTCAAGGGCTAAAGC
**R2-hisB**	TCGATTTCCGCCGATATGC
**Fup-hisB**	CTTTCGACGATGATATTGTG
**Rdown-hisB**	TCAATACGGTATCGTTCAG
**F-compl-hisB**	GAGCGAAACCTGTCCATC
**R-compl-hisB**	GGAAAGGCTTTAGCCCTTG
**F1-hisC**	AACGCACTTTCGCCTTCATG
**R1-hisC**	gtgagggcggGTAGGTTTCTGCATGTCGGTC
**F2-hisC**	agaaacctacCCGCCCTCACAGAATTTCTG
**R2-hisC**	CCTTGTTGTGCAGGCAGAC
**Famont-hisC**	CGGTTCGCATGGCCCTTG
**Raval-hisC**	CGGTGCATCCACTTCCTGAC
**F-compl-hisC**	CCCTATAGCCGCACACAG
**R-compl-hisC**	CGAAGTGGATTGTGGACATTTG
**F1-hisD**	CTGCATCATCGTGCACAAGG
**R1-hisD**	ccctcataggCCATGCGGACACTCCTTCA
**F2-hisD**	gtccgcatggCCTATGAGGGACCATGACTGC
**R2-hisD**	GGCGATTGTCGAGTGACAG
**F-compl-hisD-Xba1**	ctagtctagaTGCGCTTTTGGGCTGCAAG
**R-compl-hisD-BamH1**	acgcggatccTTGGGAACGTCGGCAGTCATG
**C184_AM_C6_F**	TTCAAGAAAACCCTCATTGCC
**C185_AM_C6_R**	CTTGCAAGCAAAATTATCAACGTA
**C186_AV_C6_F**	TACGTTGATAATTTTGCTTGCAAG
**C187_AV _C6_R**	CTTATCGATCTTCACCTGATCCG
**C251_OppA1_KpnI_F**	gactggtaccATGTATCGCAAATTTCTACTGATGG

**C252_OppA1_SacI_R**	gactgagctcTTATTTTTTAAGGGAAAGCCAACG
**C253_OppA2_KpnI_F**	cattggtaccATGGTTCGCGGAATTTTGATGACAG
**C254_OppA2_SacI_R**	gtacgagctcTTAGTTCTTGACGGATAGCCAGCG

Table S4. List of plasmids used in this study.

Plasmid name	References
**PXMCS-2 mini-Tn*5***	Sternon *et al.* 2018
**pNPTS-Δ*copA***	This study
**pNPTS-Δ*cueO***	This study
**pNPTS-Δ*hisA***	This study
**pNPTS-Δ*hisB***	Roba *et al* . 2022
**pNPTS-Δ*hisC***	This study
**pNPTS-Δ*hisD***	This study
**pMR10 *hisA***	This study
**pMR10 *hisB***	Roba *et al* . 2022
**pMR10 *hisC***	This study
**pMR10 *hisD***	This study
**pBBRMCS2-$oppA_1$**	This study
**pBBRMCS2-$oppA_2$**	This study

Table S5. List of strains used in this study.

Name	Genotype	Resistance	References
**Δ*copA***	*B. abortus* 544 Δ*copA*		This study
**Δ*cueO***	*B. abortus* 544 Δ*cueO*		This study
**Δ*hisA***	*B. abortus* 544 Δ*hisA*		This study
**Δ*hisB***	*B. abortus* 544 Δ*hisB*		Roba *et al* . 2022
**Δ*hisC***	*B. abortus* 544 Δ*hisC*		This study
**Δ*hisD***	*B. abortus* 544 Δ*hisD*		This study
**Δ*hisA* pMR10-*hisA***	*B. abortus* 544 Δ*hisA* pMR10-*hisA*	Kan^R	This study
**Δ*hisB* pMR10-*hisB***	*B. abortus* 544 Δ*hisB* pMR10-*hisB*	Kan^R	Roba *et al* . 2022
**Δ*hisC* pMR10-*hisC***	*B. abortus* 544 Δ*hisC* pMR10-*hisC*	Kan^R	This study
**Δ*hisD* pMR10-*hisD***	*B. abortus* 544 Δ*hisD* pMR10-*hisD*	Kan^R	This study
**C6**	*B. abortus* 544 Δ*hisC*		This study
**Δ*hisC*SupPA$_1$**	*B. abortus* 544 Δ*hisC*		This study
**Δ*hisC* pBBRMCS2-$oppA_1$**	*B. abortus* 544 Δ*hisC* pBBRMCS2-$oppA_1$	Kan^R	This study
**Δ*hisC* pBBRMCS2-$oppA_2$**	*B. abortus* 544 Δ*hisC* pBBRMCS2-$oppA_2$	Kan^R	This study
**pBBRMCS2**	*B. abortus* 544 pBBRMCS2	Kan^R	This study

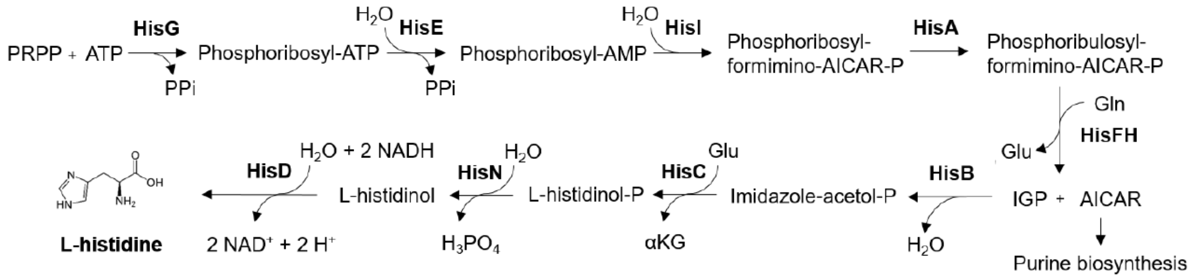

Figure S1. Schematic representation of the histidine biosynthesis pathway. HisG: ATP phosphorisobyl transferase. HisE: PR-ATP hydrolase. HisI: PR-AMP cyclohydrolase. HisA: Phosphoribosyl-formimino-AICAR-P isomerase. HisF: cyclase. HisH: Glutamine amidotransferase. HisB: imidazoleglycerol-phosphate dehydratase. HisC: Imidazole acetol phosphate aminotransferase. HisN: histidinol-phosphate-phosphatase. HisD: histidinol dehydrogenase. Abbreviations are PRPP; phosphoribosylpyrophosphate, ATP; adenosine triphosphate PPi; pyrophosphate, Gln; glutamine, Glu; glutamate, IGP: imidazole glycerol phosphate, AICAR: 5-amino-1-(5-phospho-D-ribosyl)imidazole-4-carboxamide, αKG; α-keto-glutarate, NAD; nicotinamide adenine dinucleotide.

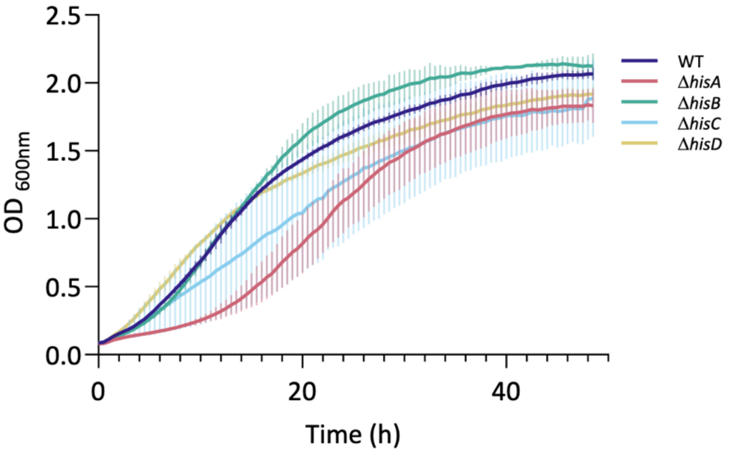

Figure S2. Histidine auxotroph mutants are able to grow in a rich medium. WT and *his* mutant strains were tested for their growth in control condition. Strains were grown in liquid TSB rich medium. OD_{600nm} was measured every 30 minutes for 48 hours. The data represent three independent replicates.

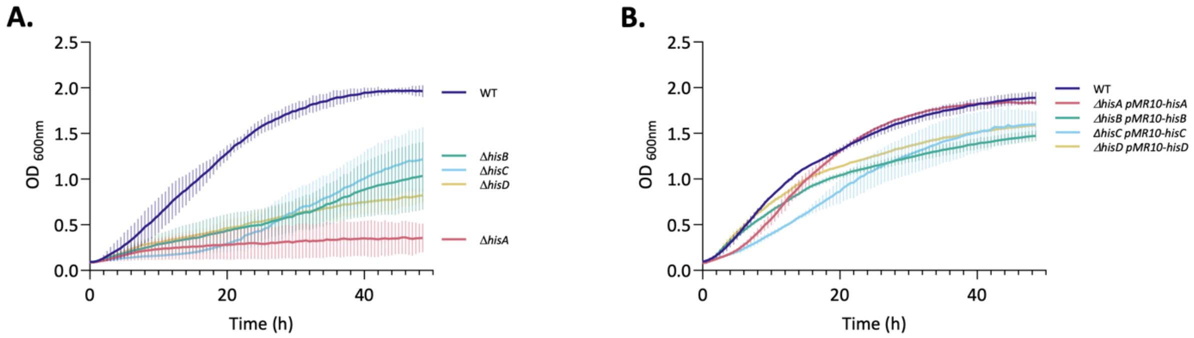

Figure S3. Histidine auxotroph mutants are copper sensitive. WT, Δ*hisA,* Δ*hisB,* Δ*hisC,* Δ*hisD,* Δ*hisA* pMR10-*hisA,* Δ*hisB* pMR10-*hisB,* Δ*hisC* pMR10-*hisC* and Δ*hisD* pMR10-*hisD* strains were tested for their sensitivity to copper toxicity for growth. **A.** WT, Δ*his* and (**B.**) complemented strains were grown in liquid TSB rich medium containing 2 mM of $CuSO_4$. OD_{600nm} was measured every 30 minutes for 48 hours. The data represent three independent replicates

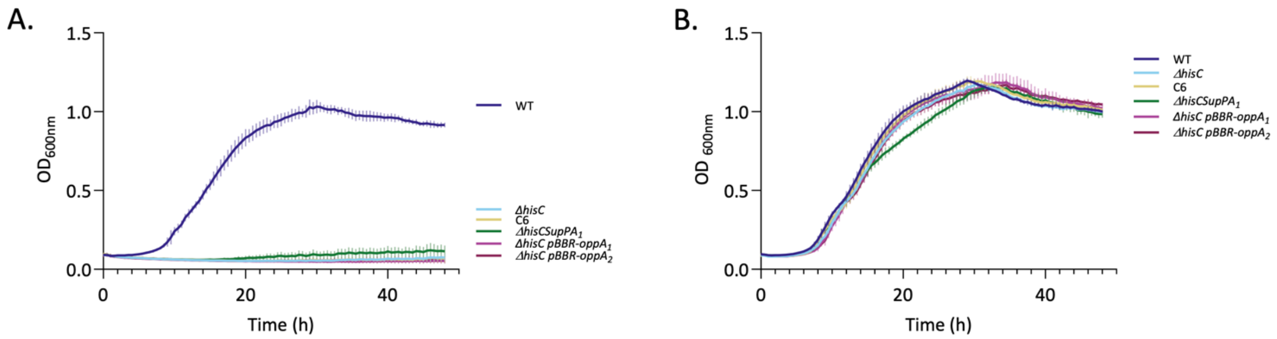

Figure S4. Suppressive mutation does not save histidine auxotrophy. WT*,* Δ*hisC* Δ*hisC*, C6, Δ*hisC*SupPA1, pBBR-*oppA*$_1$ *and* Δ*hisC* pBBR-*oppA*$_2$ strains were tested for their auxotrophy for histidine. WT, Δ*hisC* strains and derivatives were grown in liquid PE medium without (**A.**) and with 1mM histidine (**B.**). OD_{600nm} was measured every 30 minutes for 48 hours. The pMR10 is a low copy plasmid. The data represent three independent replicates.
